# Inhibitors of Nucleotide Biosynthesis as Candidates for a Wide Spectrum of Antiviral Chemotherapy

**DOI:** 10.3390/microorganisms10081631

**Published:** 2022-08-12

**Authors:** Claudia Soledad Sepúlveda, Cybele Carina García, Elsa Beatriz Damonte

**Affiliations:** 1 Laboratory of Virology, Biochemistry Department, School of Sciences, University of Buenos Aires (UBA), Ciudad Universitaria, Buenos Aires 1428, Argentina; 2 Institute of Biochemistry of the School of Sciences (IQUIBICEN), CONICET-UBA, Ciudad Universitaria, Buenos Aires 1428, Argentina

**Keywords:** wide-spectrum antiviral, host-targeted antiviral, nucleotide metabolism, *de novo* biosynthesis pathway, salvage pathway, pyrimidines, purines, DHODH inhibitors, IMPDH inhibitors

## Abstract

Emerging and re-emerging viruses have been a challenge in public health in recent decades. Host-targeted antivirals (HTA) directed at cellular molecules or pathways involved in virus multiplication represent an interesting strategy to combat viruses presently lacking effective chemotherapy. HTA could provide a wide range of agents with inhibitory activity against current and future viruses that share similar host requirements and reduce the possible selection of antiviral-resistant variants. Nucleotide metabolism is one of the more exploited host metabolic pathways as a potential antiviral target for several human viruses. This review focuses on the antiviral properties of the inhibitors of pyrimidine and purine nucleotide biosynthesis, with an emphasis on the rate-limiting enzymes dihydroorotate dehydrogenase (DHODH) and inosine monophosphate dehydrogenase (IMPDH) for which there are old and new drugs active against a broad spectrum of pathogenic viruses.

## 1. Introduction

The continuous emergence of new viruses or the re-emergence of well-known viruses with increased virulence, changes in host ranges, or altered geographic distributions have represented a serious threat to human health. This situation has been heightened in recent decades, as clearly illustrated by the H1N1 Influenza A virus (IAV) pandemic in 2009 [1], the Zika virus (ZIKV) epidemics in 2015–2016 [2], and, more recently, the explosive ongoing severe acute respiratory syndrome coronavirus 2 (SARS-CoV-2) pandemic that began in 2019 [3], as well as periodic epidemics appearing around the world due to other viruses [4]. A possible expansion of this tendency in the future is supported by evidence of the high potential of many RNA viruses to cross the barrier from animal to human, occasionally associated with efficient adaptation to the new host and human-to-human transmission [5]. Most RNA viruses are zoonotic with a natural reservoir and the chance of a spillover linked to their high rates of spread and mutation causes the appearance of new RNA viruses each year [6]. No antivirals or vaccines are currently available for emerging or re-emerging viruses, with the exception of SARS-CoV-2. With this expectation, the search for a broad-spectrum antiviral agent to combat outbreaks of viral infections is urgent.

Two different strategies are usually employed for viral chemotherapy development: the viral target-based approach, aimed at blocking a virus-encoded protein, which gives rise to the design of direct-acting antivirals (DAA), and the host-targeted approach, aimed at interfering with any cellular components involved in the virus’ multiplication cycle or pathogenesis, resulting in the design of host-targeted antivirals (HTA). Most licensed antiviral agents in current clinical use for herpesviruses, human immunodeficiency virus (HIV), hepatitis B (HBV) and C virus (HCV), or IAV are DAA [7]. Since this class of compounds targets viral proteins, they are virus-specific and offer a great chance for the selection of drug-resistant viral variants, a frequent event during the chemotherapy of RNA viruses due to their high genetic variation. In contrast, the consideration of druggable host molecules and pathways that are hijacked by viruses for productive infections as therapeutic targets has regained interest in recent years [8,9,10,11]. HTA can provide a wide range of agents with inhibitory effects against all circulating viruses that share similar host requirements as well as for future emerging or re-emerging pathogens. This strategy also allows the repurposing of drugs previously approved for other therapeutic medications with a less expensive process of antiviral development given the known information about safety, bioavailability, and the validation of compounds for clinical use [12]. Another advantage of HTA in comparison to classical DAA is a reduced challenge of selection for antiviral resistance.

The host factors analyzed as potential antiviral targets include diverse molecules and pathways such as lysosomotropic agents, lipid metabolism, protein synthesis and folding, nucleotide metabolism, ribonucleoproteins participating in the maturation and trafficking of mRNAs, kinases that modulate signaling pathways, glucosidases, and the manipulation of viral restriction factors involved in intrinsic innate immunity, among others. This review is focused on nucleotide metabolism, one of the more exploited host processes, as a potential antiviral target for several human viruses, with an emphasis on the cellular enzymes of the pathways for nucleotide biosynthesis that have been successfully tested with promising inhibitors or are considered appropriate for drug inhibition. 

## 2. Nucleotide Biosynthesis Pathways 

Pyrimidines and purines are heterocyclic metabolites with a fundamental role as the precursors for nucleic acid biosynthesis but also participate in several processes of lipid and carbohydrate metabolism, energy supply, and cofactor provision for cell survival and apoptosis as well as immune responses. There are two routes for the generation of nucleotides in mammalian cells: nucleotides can be *de novo* synthesized from small simple molecules or recycled from preformed nucleoside precursors by salvage pathways. The *de novo* synthesis presents a higher need for energy in comparison with the salvage pathway and the balance between the usage of one or other route is dependent on the cell type and the developmental state of the cells [13]. Usually, in quiescent and fully differentiated cells, the low requirement of nucleotides is supplied by the salvage pathway whereas *de novo* biosynthesis is essential in proliferating cells such as lymphocytes to satisfy their high demand for nucleic acid precursors. Furthermore, tumor cells, as well as cells infected with certain pathogenic viruses, also employ *de novo* synthesis due to their increased metabolic activity with a high requirement of nucleotides to allow cell proliferation or virus multiplication. Accordingly, high concentrations of metabolites related to *de novo* purine or pyrimidine biosynthesis have been reported in tumor cells [14,15,16] or human cytomegalovirus (HCMV)-infected cells [17], favoring the potential therapy with antimetabolites as inhibitors of nucleotide synthesis.

### 2.1. Biosynthesis of Pyrimidines

**Figure 1:**
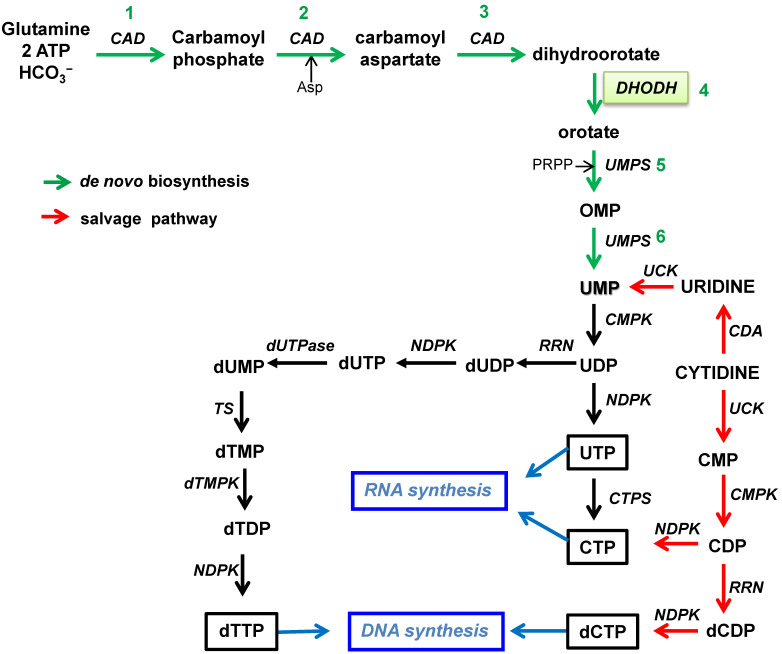
*De novo* and salvage biosynthesis pathways of pyrimidine nucleotides.

The *de novo* biosynthesis of pyrimidine nucleotides is defined as the six-step pathway leading to the production of uridine monophosphate (UMP) (Figure 1). 

The first three steps are catalyzed by the multifunctional protein called CAD, a single polypeptide that carries three enzymatic domains: carbamoyl phosphate synthase (CPS), aspartate transcarbamylase (ATC), and dihydroorotase (DHOase) [18]. Initially, CPS leads to the reaction of glutamine, bicarbonate, and ATP to produce carbamoyl phosphate, which is then converted to carbamoyl aspartate through the addition of aspartate via the enzymatic activity of ATC. In the third step, the dihydroorotase domain of CAD catalyzes the hydrolysis of carbamoyl aspartate to dihydroorotate. Next, dihydroorotate dehydrogenase (DHODH), a flavoprotein localized in the mitochondrial membrane, oxidizes dihydroorotate to orotate transferring two electrons to the respiratory chain via ubiquinone [19]. In steps 5 and 6 of the pathways, the UMP synthase (UMPS) is a bifunctional protein acting for the transformation of orotate into UMP after two consecutive reactions. Orotate is converted first into orotidine monophosphate (OMP) through the transfer of the phosphoribosyl group of 5-phosphoribosyl-1-pyrophosphate (PRPP) by the orotate phosphoribosyltransferase located in the N-terminal domain of UMPS, and then the OMP decarboxylase present in the C-terminal domain converts OMP into UMP [20]. As observed in Figure 1, UMP is the central product of the *de novo* pyrimidine pathway and a key substrate for nucleic acid biosynthesis since it can provide pyrimidine ribonucleotides and deoxyribonucleotides conforming to RNA and DNA, respectively. UMP can be transformed into UDP and UTP by cytidine monophosphate kinase (CMPK) and nucleoside diphosphate kinase (NDPK), respectively, and then, alternatively, CTP synthase can convert UTP in CTP to assess the synthesis of the two pyrimidine ribonucleotides. On the other hand, for DNA synthesis, UDP is first deoxygenated into deoxy-UDP (dUDP) by ribonucleotide reductase (RNR) and later phosphorylated by NDPK. Then, dUTP is rapidly transformed by dUTPase into dUMP. The enzyme thymidylate synthase (TS) catalyzes the conversion of dUMP into dTMP, which can be phosphorylated by the corresponding kinases into dTTP.

Concomitantly with *de novo* biosynthesis, pyrimidine nucleotides can be obtained by salvaging products from the intracellular catabolism of nucleic acids or the pool of extracellular nucleosides internalized into the cell through specific transport channels and pumps (Figure 1). Uridine or cytidine can be phosphorylated to UMP via uridine cytidine kinase (UCK) or cytidine deaminase (CDA) and UCK, respectively. Uridine is the predominant extracellular nucleoside circulating in mammalian plasma and, consequently, is the main source for the utilization of the salvage pathway generating UMP. Alternatively, cytidine can be converted to cytidine diphosphate (CDP) via UCK and CMPK. Two branches open from CDP: it can be either further phosphorylated to CTP by NDPK or deoxygenated to dCDP by RNR and then phosphorylated to dCTP, completing the total pyrimidine nucleotides required for nucleic acids in the cell.

### 2.2. Biosynthesis of Purines

**Figure 2:**
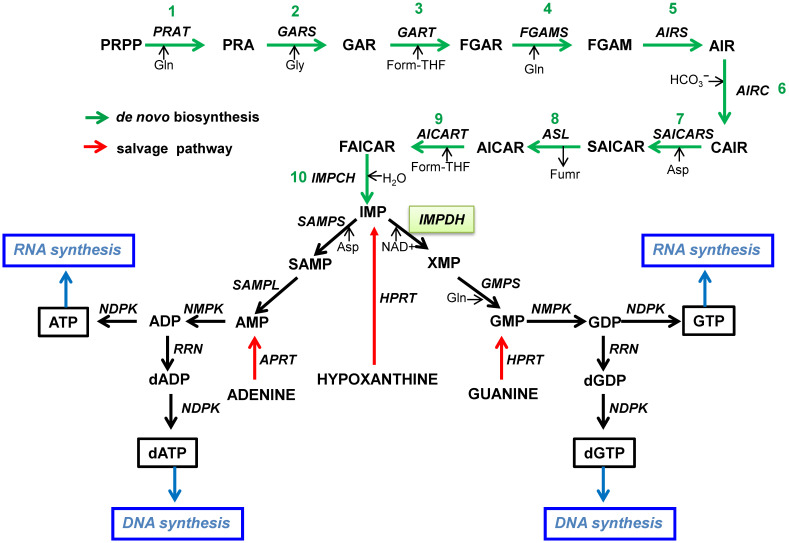
*De novo* and salvage biosynthesis pathways of purine nucleotides.

The *de novo* synthesis pathway of purines is a complex pathway consisting of 10 consecutive, regulated steps to produce inosine monophosphate (IMP) from the initial substrate PRPP (Figure 2) [21]. In the first step, the phosphoribosylamidotransferase (PRAT) catalyzes the transfer of an amido group from glutamine to PRPP to obtain phosphoribosylamine (PRA). Here, a difference between the purine and pyrimidine metabolic pathways can be seen; in purines, ribosyl phosphate is bound from the beginning of the pathway (step 1), whereas in pyrimidines, it is incorporated after the formation of the heterocycle ring in orotate (step 5). In step 2, PRA reacts with glycine to form glycineamideribonucleotide (GAR) with the catalysis of GAR synthase (GARS). Next, GAR transformylase (GART) catalyzes the transfer of the formyl group from formyltetrahydrofolate (THF) to produce formyl GAR (FGAR), which is then converted (step 4) to formylglycineamidineribonucleotide (FGAM) catalyzed by FGAM synthase (FGAMS). Step 5 consists of the cyclization of FGAM to 5-aminoimidazole ribonucleotide (AIR) catalyzed by AIR synthase (AIRS), and next (step 6), a carboxyl is introduced to AIR-by-AIR carboxylase generating AIR carboxamide (CAIR). In step 7, the reaction of CAIR with aspartate is catalyzed by N-succino-5-aminoimidazole-4-carboxamide ribonucleotide synthase (SAICARS) to obtain SAICAR. In step 8, adenylosuccinate lyase (ASL) eliminates fumarate from SAICAR to produce AICAR, which in step 9 is then converted to 5-formyl-AICAR (FAICAR) through the transfer of the formyl group from formyl THF catalyzed by AICAR transformylase (AICART). Finally, in step 10, IMP cyclohydrolase (IMPCH) catalyzes an intramolecular ring closure to generate IMP.

Alternatively, the purines adenine, guanine, and hypoxanthine can be transformed into their nucleoside monophosphates through the salvage pathways with the consumption of PRPP and catalyzed by the respective phosphoribosyltransferase (Figure 2).

As a UMP in pyrimidine nucleotide synthesis, IMP is considered the key product of the *de novo* purine pathway since IMP is the source of all the purine nucleotides for nucleic acid synthesis. To this end, IMP is subsequently either converted to adenosine monophosphate (AMP) or guanosine monophosphate (GMP) in reactions catalyzed by specific enzymes for each nucleoside. On the one hand, IMP is converted to adenylosuccinate (SAMP) with the catalysis of SAMP synthase, and then SAMP lyase (SAMPL) cleaves SAMP to AMP and fumarate. On the other hand, IMP dehydrogenase (IMPDH) catalyzes the nicotine-adenine dinucleotide (NAD+)-dependent oxidation of IMP to xanthine monophosphate (XMP), which is then aminated to GMP-by-GMP synthase (GMPS). Later, the corresponding nucleotides are obtained by phosphorylation with the respective kinases.

## 3. Inhibition of Nucleotide Biosynthesis and the Potential for Therapy

The inhibition of the purine and pyrimidine pathways of biosynthesis, shown in Figure 1 and Figure 2, at any particular step results in the depletion of the metabolites of the subsequent points in the route and the eventual inhibition of RNA or DNA synthesis as well as alterations in all the metabolic reactions where nucleotides participate. To optimize this inhibition, selected targets have been chosen as potential strategies to treat diverse human diseases affected by nucleic acid metabolism.

For pyrimidines, the conversion of dihydroorotate to orotate is the rate-limiting step in the *de novo* pathway and, consequently, DHODH is the most attractive and privileged drug target to inhibit this route of synthesis [22]. DHODH is unique in performing this reaction and, additionally, the oxidoreductase activity of DHODH links the nucleotides with the mitochondrial respiratory chain and oxygen consumption. Interestingly, DHODH inhibitors have been approved by the United States Food and Drug Administration (FDA) and are employed for the therapy of autoimmune diseases such as rheumatoid arthritis and multiple sclerosis [23,24] and they are also the objects of active research in cancers [25,26], parasites [27], and virus infections [28].

In virus infections, their diverse properties have been considered responsible for the antiviral activity of DHODH inhibitors. First, virus multiplication is mainly affected due to the lack of pyrimidines in treated infected cells with the consequent blockade in viral genome replication and virion production. Besides their role as the providers of the precursors for viral RNA or DNA genes, nucleotides are also functional in cell signaling. Therefore, it was demonstrated that nucleotide deprivation is also related to innate immune responses because the presence of pyrimidine inhibitors induced an improved expression of cellular antiviral genes in virus-infected cells. However, the mode of this interaction between pyrimidine biosynthesis and host innate immunity is not yet fully elucidated and has produced contrasting experimental results. In fact, it has been reported that DHODH inhibitors can establish an antiviral state in host cells through the activation of interferon (IFN)-stimulated gene (ISG) expression mediated either by IFN-dependent [29,30] or non-canonical IFN-independent mechanisms [31,32,33]. An additional antiviral effect associated with DHODH inhibition is the anti-inflammatory activity triggered by the reduction in pathogenic cytokines usually overexpressed in severe virus infections [34,35]. Altogether, the combination of these three possible mechanisms warrants the susceptibility of a wide spectrum of viruses to DHODH inhibition.

CAD, the multienzymatic protein that initiates the *de novo* synthesis of pyrimidines, as well as enzymes acting in steps downstream of DHODH activity such as UMP or CTP synthase, is also a target for antiviral research but with less success than the DHODH inhibitors, probably due to a lower index of selectivity [36]. Furthermore, inhibitors of the salvage pathway have also been reported for several RNA viruses [37]. However, their antiviral chemotherapeutic perspectives do not seem comparable to the *de novo* biosynthesis inhibitors given the demand for a large intracellular nucleotide pool by replicating viruses, meaning that *de novo* synthesis is more critical during virus infection than the salvage route.

For purines, a similar election of selective targets as described for pyrimidines was observed. IMPDH is the rate-limiting enzyme for the *de novo* synthesis of the guanosine nucleotides required for DNA or RNA replication and the main target for the blockade of purine biosynthesis [38,39]. Two isoforms of human IMPDH possessing 84% sequence homology have been identified: type I IMPDH is constitutively expressed and is the major species found in normal cells, whereas type II is found at very low levels in most resting cells but its expression is upregulated in cancer and replicating cells such as activated lymphocytes [40]. Therefore, the selective inhibition of type II IMPDH could provide therapeutic advantages in pathological conditions and, similar to DHODH inhibitors, IMPDH inhibitors have demonstrated a wide spectrum of anticancer [41], immunosuppressive [42], and antiviral activity [43]. There are also examples of IMPDH inhibitors, which after clinical trials, were licensed for immunosuppressive therapies, particularly in transplant recipients [44,45]. Consequently, the main clinical use of IMPDH inhibitors is as immunosuppressants to prevent graft rejection. The strong immunosuppressive properties, due to the depletion of purine nucleotides, could hamper the use of IMPDH inhibitors in viral diseases related to virus-triggered immunological events as well as in the adaptive immune response for virus clearance. However, the combination of the immunosuppressive and virus-modulating activities of these drugs could provide adequate tools for managing the treatment of transplant patients at risk of certain opportunistic virus infections [46,47].

## 4. Pyrimidine Inhibitors as Antivirals

At present, an important number of pyrimidine inhibitors have been shown to improve viral infection outcomes both *in vitro* and *in vivo* [48]. One particular aspect of all the reported human DHODH inhibitors is that they target the ubiquinone-binding site in the N-terminal domain of DHODH. In particular, residues (aa 30–68) inside the ubiquinone-binding pocket have been identified as critical for site interactions, and efforts to develop potent DHODH inhibitors have been focused on that area [28]. It is worth mentioning that DHODH inhibitors are likely to have additional targets besides DHODH, promoting anti-kinase effects, or acting as aryl hydrocarbon partial antagonists. The antiviral efficacy and current clinical applications of several compounds that have been approved or are in the experimental phase are detailed in this section. 

### 4.1. Leflunomide and Teriflunomide

Leflunomide, a monocarboxylic acid amide derivative of isoxazole, is an immunosuppressive agent that was approved by the FDA in 1998 to treat active moderate-to-severe rheumatoid and psoriatic arthritis [49]. This drug is metabolized into teriflunomide, its active metabolite also known as A77 1726, which inhibits DHODH activity through its malononitrile open ring by non-competitive binding to ubiquinone [50,51]. Teriflunomide was approved in 2012 by the FDA for the treatment of multiple sclerosis [52] making it an attractive candidate for alternative uses having already overcome regulatory barriers. Both compounds present several antiviral activities *in vitro*; however, it remains controversial and to be determined whether their main mechanism of virus replication inhibition is the interference of pyrimidine nucleotide synthesis.

Initial reports showed the leflunomide inhibition of HCMV and polyoma BK virus (BKV) replication [53,54]. Later, it was reported that teriflunomide inhibits cellular proliferation and promotes apoptosis in Epstein–Barr virus (EBV)-transformed B cells [55]. Also, it was observed that teriflunomide inhibited the development of EBV-induced lymphomas in a humanized mouse model and a xenograft model and blocked a lytic EBV infection *in vitro* by impairing the initial steps of lytic viral reactivation and by suppressing viral DNA replication [55]. Leflunomide is being increasingly used to treat different human herpesviruses in organ transplant recipients [56].

More recently, it was demonstrated that teriflunomide interferes with the replication of different strains of the hemorrhagic fever Junín arenavirus (JUNV). Notably, it was observed that the combined treatment of teriflunomide and ribavirin had significantly more potent antiviral activity against JUNV than each single drug treatment. Mechanistic studies showed that this antiviral activity was reversed by the exogenous addition of uridine or orotic acid [57]. 

Subsequently, it was demonstrated that the use of leflunomide mitigated weight loss, reduced the viral load in the lungs, and prolonged the survival time in IAV (H5N1 or H1N1)-infected mouse models [58], but viral inhibition was achieved by blocking the activity of Janus kinase 1 (JAK1) and JAK3. Two previous studies from the same group showed that the reduction in alveolar fluid clearance, a pathophysiologic sequela of respiratory syncytial virus (RSV) infection, could be prevented by leflunomide and teriflunomide. Importantly, the drug effects could be reversed by exogenous uridine suggesting that the impairment of DHODH enzymatic activity was the main antiviral mechanism involved [59,60]. 

Importantly, leflunomide and teriflunomide showed strong anti-SARS-CoV-2 activity *in vitro*, with considerably higher effectiveness than treatment with favipiravir [34], highlighting the relevance of the immunosuppressive potency of these drugs in controlling this viral infection. In fact, leflunomide was tested in a clinical trial for COVID-19 therapy at the People’s Hospital of Wuhan University, China. The results from a compassionate-use study showed that the shedding time of patients treated with leflunomide (median 5 days) was significantly shorter than that of control patients (median 11 days). Additionally, C-reactive protein levels were reduced in leflunomide-treated patients, confirming the dual antiviral and anti-inflammatory activity of leflunomide [61]. However, in another study, COVID-19 patients with prolonged PCR positivity showed no benefit in terms of the duration of viral shedding with the combined treatment of leflunomide and IFN-α-2a in comparison with IFN-α-2a alone [62]. To further test the efficacy of leflunomide for SARS-CoV-2 therapy, another ongoing study is specifically analyzing the outcomes for a loading dose of leflunomide of 100 mg daily for three days, followed by leflunomide 10–20 mg daily, to complete a total treatment of 10 days, versus the standard of care (https://www.lifearc.org/news/covid-19-information/covid-19-funding/defeat-covid-study/, accessed on 26 July 2022). 

### 4.2. Brequinar (DuP-785)

Brequinar is a 4-quinoline carboxylic acid derivative with known immunosuppressive and anti-proliferative activities that has been evaluated in multiple clinical trials as a potential treatment for cancer [25,63]. This compound is a potent DHODH inhibitor within nanomolar range concentrations that competitively binds to ubiquinone and disrupts the catalytic cycle of DHODH [50,64]. In the last decade, several antiviral activities of brequinar against important human RNA viral pathogens were reported as a single treatment or combined with other compounds. In most of these studies, the addition of exogenous uridine reversed its antiviral activity, indicating that the antiviral effect of brequinar could be attributed to the alteration of pyrimidine synthesis. The viral experimental models where brequinar has been tested and reached positive results include several flaviviruses, such as West Nile (WNV), yellow fever virus (YFV), dengue virus (DENV), and ZIKV [65,66], other important pathogenic viruses, such as Ebola (EBOV) [31], HCMV [67], and IAV, and different enteroviruses such as EV71, EV70, and coxsackievirus B3 (CVB3) [68]. Moreover, this drug and the above-described leflunomide robustly inhibited the replication of laboratory and clinical strains of rotavirus in human intestinal Caco2 cell lines as well as in human primary intestinal organoids [69]. 

For SARS-CoV-2 *in vitro* infection, it was demonstrated that brequinar was effective at sub-micromolar concentrations [34]. Moreover, in a model of BALB/c mice infected with the SARS-CoV-2 Beta strain, the combined treatment of brequinar and molnupiravir (DAA) significantly reduced viral titers and pathology in comparison to the use of monupiravir alone [70]. Indeed, phase I randomized (NCT04425252) and phase II (NCT04575038) clinical trials were started at the end of 2020 to evaluate the effects of brequinar in hospitalized patients with COVID-19 infections.

### 4.3. S312 and S416

Two thiazole derivatives endowed with unique binding characteristics in the ubiquinone-binding pocket of human DHODH [71] were selected for their high efficiency and low toxicity among 280,000 compounds screened in hierarchical structure analysis. The DHODH inhibitors, identified as S312 and S416, with previously shown favorable drug-like and pharmacokinetic profiles [72] presented broad-spectrum antiviral effects against various RNA viruses in different cell models. The antiviral activities of these inhibitors were reported against different strains of IAV, ZIKV, EBOV, and SARS-CoV-2, with low effectiveness in *in vitro* concentration values ranging from 7.5 nM to 1.56 µM [34]. Notably, *in vivo* experiments showed that S312 efficacies were superior to oseltamivir (a DAA) in treating the late infection phase and reducing cytokine and chemokine storms in IAV-infected mice because of its dual antiviral and immune regulation activities. In addition, combined with oseltamivir, S312 conferred an additional 16.7% survival in the severe, late infection stage [34].

### 4.4. Emvododstat (PTC299)

PTC299 is an orally bioavailable small molecule that inhibits DHODH at concentrations in the nanomolar order [73]. PTC299 has favorable drug properties targeting hematological tumors and normalizes vascular endothelial growth factor (VEGF) levels in cancer patients [74]. A study by Luban et al. demonstrated that PTC299 inhibited SARS-CoV-2 replication in Vero E6 cells. Furthermore, researchers also showed that PTC299 had broad-spectrum *in vitro* antiviral activity against viruses such as EBOV, poliovirus, HCV, and Rift Valley fever virus (RVFV). Notably, the inhibition of viral replication was lost by the addition of exogenous uridine, confirming PTC299 antiviral activity as a DHODH inhibitor. In particular, it was shown that PTC299 inhibited the production of several interleukins and VEGF in tissue culture models (35). The combination of antiviral broad activity, cytokine inhibitory activity, and previously established favorable pharmacokinetic and human safety profiles render PTC299 a promising therapeutic for COVID-19 and it is currently being evaluated in a phase II/III study, PTC299-VIR-015-COV19 (FITE19) (https://clinicaltrials.gov/ct2/show/NCT04439071, accessed on 26 July 2022).

### 4.5. Vidofludimus Calcium (IMU-838)

IMU-838, also known as vidofludimus calcium, was initially proposed as a therapeutic for multiple sclerosis [75,76]. IMU-838 is an oral selective immunomodulator that inhibits the intracellular metabolism of activated immune cells by blocking DHODH activity at low nanomolar concentration ranges [75] and is almost three times more potent in inhibiting dihydroorotate oxidation in comparison to teriflunomide. It has been proven that IMU-838 presents high, broad-spectrum *in vitro* antiviral activity against HCMV, HIV-1, HCV, and SARS-CoV-2. Notably, in SARS-CoV-2-infected cells, the combined treatment with IMU-838 and remdesivir (a DAA) provoked a total inhibition of viral yield, suggesting that the combination of a DHODH inhibitor and DAA was an effective antiviral strategy [77]. Currently, IMU-838 is being evaluated for its therapeutic effect as a COVID-19 therapy in a phase II clinical trial (https://clinicaltrials.gov/ct2/show/NCT04379271, accessed on 26 July 2022).

### 4.6. A3

The small-molecular-weight pyrimidine biosynthesis inhibitor identified as A3 was found to be active against IAV after the screening of approximately 61,600 molecules. This compound showed *in vitro* low toxicity and excellent antiviral activity at the micromolar concentration range [78]. In fact, better results were found in primary human tracheal-bronchial epithelial cells, demonstrating a strong effect on viral replication. In this study model, the A3 inhibition of virus polymerase function and RNA synthesis was reversed with the addition of uracil or orotic acid, demonstrating that the antiviral mechanism of action involves the inhibition of DHODH. The same group of researchers reported the broad-spectrum antiviral activity of this compound, including different families of RNA (*Orthomyxoviridae, Paramyxoviridae, Rhabdoviridae, Togaviridae, Flaviviridae*), DNA (*Poxviridae* and *Adenoviridae*), and retroviruses, though with varying effectiveness [78]. Later, another report also identified A3 as an effective antiviral drug against arenaviruses, further demonstrating its ability to block viral RNA replication and transcription. Moreover, the same study suggested that A3 can induce random mutations in the viral RNA that decrease the infectivity of the virus [79].

### 4.7. FA-613

After screening 50,240 compounds that target the influenza virus nucleoprotein, the compound identified as FA-613 was found to be effective against IAV and influenza B (IBV) viruses [30]. When BALB/c mice were challenged with IAV H1N1, FA-613 treatment for 3 days protected 30.7% of the mice from death. Its effects were not limited to the inhibition of RNA replication since FA-613 also triggered host innate immunity. The survival of mice infected with H1N1 was prolonged by treatment with FA-613 and the expression of different ISG was triggered [30]. Complementary studies from the same group indicated that FA-613 had broad-spectrum antiviral efficacy, presenting positive results against highly pathogenic IAV, EVA71, RSV, SARS-CoV-1, Middle East respiratory syndrome-related coronavirus (MERS-CoV), and human rhinovirus A. Interestingly, FA-613 lost its antiviral potency in the IFN-deficient Vero cell line while maintaining its inhibitory activity in an IFN-competent cell line that showed elevated expression of ISG when infected in the presence of FA-613. Notably, the antiviral effect of FA-613 was reversed by the addition of exogenous uridine or orotic acid, suggesting that the main mechanism of action could target DHODH; however, direct DHODH inhibition by FA-613 is unclear [30].

### 4.8. BAY2402234

Mathieu et al. screened 492 compounds inhibiting SARS-CoV-2 replication and found BAY2402234, a novel, potent selective DHODH inhibitor that blocked almost 100% of SARS-CoV-2 particle production at 0.6 µM [80,81]. In addition, the combination of teriflunomide, IMU-838/vidofludimus, and BAY2402234 inhibited SARS-CoV-2 replication and reduced viral yield by at least two orders of magnitude in Vero E6 and Calu-3 cells infected with the Alpha and the Beta variants of SARS-CoV-2 [70].

### 4.9. MEDS433

MEDS433 is a new generation of DHODH inhibitor with a very low effective concentration of around 1.2 nM for human DHODH [82]. It was shown that MEDS433 inhibited herpes simplex virus types 1 and 2 (HSV-1 and 2) *in vitro* and displayed highly synergistic antiviral activity when used in combination with acyclovir (a DAA) [83]. This study of the HSV replication cycle in cells treated with MEDS433 revealed that it prevented the accumulation of viral genomes and reduced late gene expression, thus suggesting an impairment at a stage prior to viral DNA replication consistent with the ability of MEDS433 to inhibit DHODH activity. 

In fact, the anti-HSV activity of MEDS433 was abrogated by the addition of exogenous uridine or orotate. A combination of MEDS433 and dipyridamole (DPY), an inhibitor of the pyrimidine salvage pathway, was then observed to be effective in inhibiting HSV replication even in the presence of exogenous uridine, thus mimicking *in vivo* conditions. Importantly, when combined with acyclovir and DPY in checkerboard experiments, MEDS433 exhibited highly synergistic antiviral activity. These findings suggest that MEDS433 is a promising candidate as either a single agent or in combination with existing DAA to develop new strategies for the treatment of HSV infections. In addition to HSV, MEDS433’s effectiveness against respiratory viruses, such as IAV and RSV, has been reported. Importantly, it was demonstrated that MEDS433 inhibited the *in vitro* replication of different coronaviruses such as HCoV-OC43 and HCoV-229E, including SARS-CoV-2, in Vero E6 and Calu-3 cells at the nanomolar range with low toxicity [84], denoting the good prospects of this compound as a wide-ranging antiviral therapeutic option.

### 4.10. RYL-634

The quinolone-derived RYL-634 is the result of the screening of a 200 biaryl-substituted quinolone library [85]. This compound showed an excellent antiviral effect against DENV, ZIKV, EV71, and HIV at nanomolar concentration doses, and a significant inhibition against Chikungunya virus (CHIKV), severe fever with thrombocytopenia syndrome virus (SFTSV), RSV, IAV, and MERS-CoV at submicromolar concentrations. Additionally, RYL-634 efficiently inhibited EBOV infection in the VP30-based trans complementation EBOV cell culture system with an efficacy comparable to that of remdesivir (DAA). The antiviral effect of RYL-634 was also observed in VeroE6-VP30 cells, excluding the cell-type-specific antiviral effects [86]. The exogenous addition of different metabolites in the pyrimidine *de novo* synthesis pathway confirmed DHODH as the target of RYL-687. These data provide evidence that such quinolone-derived compounds are promising therapeutic candidates for viral infections.

### 4.11. GSK 983

An EBOV minigenome assay was used to screen 200,000 compounds for inhibition of the viral polymerase complex and several compounds with an amino-tetrahydro carbazole scaffold were selected. In particular, the compound identified as SW835, structurally similar to GSK983, a DHODH inhibitor known to have broad-spectrum antiviral activity, was characterized and showed potent inhibition of the replication of EBOV and ZIKV. In addition, nucleoside and deoxynucleoside studies demonstrated that the depletion of pyrimidine pools contributes to its antiviral activity. Moreover, SW835 induced the expression of transcription factor IRF1, which is required for ISG induction. Therefore, the inhibition of the DHODH pathway activates an IRF1-dependent innate immune response that subverts viral immune evasion functions [31].

### 4.12. AR-12 Derivatives

The celecoxib-derived anticancer agent AR-12 with antiviral activity against a broad range of viruses was used as a model to develop additional antiviral agents, such as the AR-12 derivatives P12-23 and P12-34. These compounds were effective in suppressing DENV, ZIKV, and Japanese encephalitis virus (JEV) replication at nanomolar concentrations, which represents a 10-fold improvement in the efficacy and selectivity indices over AR-12. The antiviral activity of both P12-23 and P12-34 consists of the inhibition of viral RNA replication, with no effect on viral binding, entry, or translation. Interestingly, the antiviral activity of AR-12 and its derivatives was reversed by the addition of exogenous uridine or orotate. Animal tests with mice showed that P12-34 significantly improved their survival after subcutaneous challenge with DENV, turning this compound into a suitable candidate for clinical evaluation as a therapeutic to control flaviviral outbreaks [87].

The efficacy and antiviral spectrum of the DHODH inhibitors discussed here are summarized in Table 1.

## 5. Purine Inhibitors as Antivirals

The correct balance of purine is essential for nucleic acids, coenzymes, cell proliferation, cell signaling, and energy transfer molecules. IMPDH inhibition elicits a variety of biological responses, which is why this enzyme has emerged as an important target for antiviral, antileukemic, and immunosuppressive therapies. IMPDH inhibitors were divided into three groups according to the binding site and the competition with the natural ligands IMP and NAD+ [88]. It was found that one group competes for the position of the natural IMP substrate and includes ribavirin (1-β-D-ribofuranosyl-1,2,4-triazole-3-carboxamide), mizoribine (4-carbamoyl-1-β-D-ribofuranosyl-imidazolium-5-olate), and EICAR (5-ethynyl-1-β-D-ribofuranosylimidazole-4-carboxamide); another group competes for the NAD+/NADH cofactor binding site, including TAD (thiazole-4-carboxamide adenine dinucleotide) and its analogues; and the third group binds at an allosteric site when the enzymatic complex is formed similar to mycophenolic acid and VX-497 [89].

In combined therapy, IMPDH inhibitors also potentiate the antiviral activity of purine nucleoside analogs through more efficient phosphorylation arising from the depletion of dGTP stores and increased pool phosphate donor IMP by IMPDH inhibition [90,91]. 

### 5.1. Ribavirin

Ribavirin (RIB, Virazole^®^, Valeant Pharmaceuticals, Laval, QC, Canada) was the first synthetic nucleoside analog with broad-spectrum antiviral activity against DNA and RNA viruses [92]. There are several proposed mechanisms of antiviral action acting in different ways on different viruses [93]. The first mechanism proposed is the inhibition of IMPDH through competition with the IMP of RIB monophosphate, decreasing the reserves of guanosine nucleosides [94]. Second, immunomodulatory activity increases the activity of T lymphocytes, especially Th [95], or affects the cytokine profile [96]. Other mechanisms involve the inhibition of cap incorporation into RNA [97], direct inhibition of viral polymerase [98], and lethal mutagenic effect due to the incorporation into the viral genome of its triphosphate as an analog of GTP [99]. The incorporation of this unnatural nucleotide into viral RNA catalyzed by viral RNA-dependent RNA polymerase is further amplified by the reduced GTP pools caused by IMPDH inhibition, increasing the frequency of RIB triphosphate incorporation into the GTP locus [100,101]. 

Although RIB was active *in vitro* against a broad spectrum of viruses, it did not correlate to benefit in *in vivo* assays in many cases and its clinical use is only formally approved for the treatment of RSV infections [102] and HCV combined with INFα [103]. Although not approved, it is also the indicated treatment for infections with viruses that cause hemorrhagic fever [104]. Currently, several studies are underway where its clinical use is proposed against influenza [105], viruses that cause hemorrhagic fevers or emerging viruses [106,107], and many others where it is suggested that the effectiveness should be reviewed [108]. 

RIB showed synergistic effects when used in combination with other antiviral compounds, such as with entecavir against wild-type and lamivudine-resistant HBV [109], 2′,3′-dideoxyinosine against HIV [110], PM-523 against IAV [111], or acyclovir against HSV-1 [112], and even showed antagonism when combined with other nucleosides [113,114]. Triple therapy regimens with telaprevir, boceprevir, or sofosbuvir, plus pegylated IFNα (PegIFNα) gained approval in 2011, with the success of this approach due to RIB’s ability to prevent the relapse or emergence of high- and low-grade resistance to HCV protease inhibitors [115].

### 5.2. Mizoribine

Mizoribine (MZB, Bredinin^®^, Asahi Kasei Pharma Corporation, Tokyo, Japan) is an imidazole nucleoside-based immunosuppressive agent clinically approved in Japan since 1984. Its active form requires phosphorylation by adenosine kinase (AK) to mizoribine-5‘-monophosphate (MZB-P) that competitively inhibits GMP synthesis by blocking the IMP binding site to IMPDH and also secondarily inhibiting GMPS [116].

Furthermore, MZB possesses *in vitro* antiviral activity against IAV and IBV, RSV, HSV, and HCMV [117,118,119]. As occurs with several powerful immunosuppressants, its combination with other licensed drugs produces an extra antiviral benefit in transplant patients [120] or inflammatory diseases associated with viral infections since it increases the efficacy of glucocorticoids, reduces the viral replication induced by the long-term use of high-dose glucocorticoids, and has fewer adverse effects than other drugs [121].

During the SARS-CoV-2 pandemic, it was possible to associate a greater severity of the disease with hypertensive patients; a possible explanation is the increased plasma concentration of the heat-shock protein 60 (HSP60) in these patients. Through its canonical damage-associated molecular pattern characteristics via toll-like receptors, HSP60 activates the NF-κB pathway, stimulating the production and release of proinflammatory cytokines. HSP60 inhibitors, such as MZB [122], could be beneficial agents for hypertensive patients infected with SARS-CoV-2. Moreover, MZB has also shown antiviral activity against SARS-CoV-1 [123].

### 5.3. EICAR

EICAR was reported in 1988 as a potent antileukemic agent and, given its similar structure to RIB, was quickly evaluated as an antiviral showing a similar spectrum of action but with a 10–100 times higher inhibitory potency only associated with the depletion of guanine nucleotides [124,125,126]. Cellular AK is responsible for the conversion to EICAR 5′-monophosphate, which competes with IMP and causes the rapid and irreversible inhibition of IMPDH [127].

Although *in vivo* antiviral assays have been reported [128], this compound was not widely accepted in the clinic, but it was the basic structure for the synthesis of multiple analogs and derivatives still in the experimental phase [129,130].

### 5.4. C-Nucleosides

Pyrazofurin was the first known C-nucleoside but never had a chance to be marketed as an antiviral. Its structure heralded it as a powerful broad-spectrum antiviral and it was a molecule that gave rise to a huge number of compounds also evaluated for antiviral activity such as thiazofurin [131].

Thiazofurin (2-β-D-ribofuranosylthiazole-4-carboxamide) inhibits IMPDH after metabolic activation by phosphorylation and conversion to TAD [132], an analog of NAD that mimics the natural cofactor, and very potently inhibits IMPDH leading to decreased GTP concentration. Its initial trials as an antiviral compound date back to 1977 when C-glycosylthiazoles structurally related to RIB were studied *in vitro* against HSV-1, parainfluenza virus (HPIV-3), and rhinovirus (HRV-13) [133,134] and later against a wide variety of RNA and DNA viruses [135]. These C-nucleosides have the advantage over N-nucleosides of being resistant to degradation by cellular phosphorylase. Various derivatives equally as or more powerful than the original were developed [135,136]. Interest in C-nucleosides has recently been renewed with the appearance of two new compounds: BCX4430, effective against EBOV infections [137], and GS-6620 for the treatment of HCV infections [138], but with other targets for their mechanisms of action.

### 5.5. Mycophenolic Acid

Mycophenolic acid (MPA) is a reversible and selective inhibitor of IMPDH of non-competitive action with respect to IMP as well as NAD because its binding to IMPDH occurs after the formation of the IMPDH-IMP-NAD ternary complex [43]. It is prescribed as a formulation of mycophenolate mofetil (MMF, CellCept^®^ Roche Laboratories, Palo Alto, CA, USA), a prodrug using the morpholino ethyl ester of MPA designed to overcome the low oral bioavailability of MPA [139]. MPA was tested as a broad-spectrum antiviral shortly after its discovery in 1968 [140]. Since then, the antiviral action of MPA has been evaluated against various human, animal, and plant viruses with a variety of genome types [141,142]. In the last decade, the reports correspond to its *in vitro* evaluation against emerging viruses [143,144,145,146] or against viruses that sporadically re-emerge generating major local outbreaks [147,148,149,150,151]. Moreover, several reports of cell-based high-throughput screening assays were also published where MPA was evaluated using IAV and IBV [152], vaccinia (VACV) [153], norovirus (NV) [47], hepatitis E (HEV) [154], and MERS-CoV [145] viral pseudoparticles. In all those cases, MPA showed a potent antiviral effect through the inhibition of the *de novo* biosynthesis of nucleic acids. 

In other cases, it was possible to demonstrate the accessory mechanisms of antiviral action associated with purine depression. For instance, MMF induces apoptosis in several cancer lines [155]. However, the inhibition of flavivirus-induced apoptosis by MMF was observed because the depletion of GTP prevented RNA genome replication and the production of viral progeny and, therefore, the viral cytopathic effect [65].

Another cellular mechanism usually induced by viruses for their multiplication is autophagy. Three autophagy-related genes, ATG3, ATG5, and ATG7, were verified to be downregulated by MPA treatment [156] and caused the acceleration of autophagosome degradation by HPRT, an enzyme involved in the pathway of purine salvage, which is upregulated by IMPDH inhibition in the *de novo* pathway [157].

Viral targets were also proposed for MPA, such as anti-MERS-CoV and SARS-CoV-2 activity, due to the inhibition of papain-like protease (PLpro), which is responsible for processing nonstructural viral polyproteins for maturation [146,158]. MERS-CoV PLpro can deubiquitinate INF regulatory factor 3, preventing its translocation to the nucleus and thus producing a suppression of INF β production; the results are the innate immune suppression of host cells. 

A frequent complication after transplantation is the development of malignant neoplasms due to infection and clonal expansion of oncogenic viruses as a result of chronic immunosuppression. When MMF was used as part of immunosuppression, a decreased incidence of malignancies and risk of death was observed [159]. Patients with chronic infection who need to be transplanted could benefit from treatment with MMF since, in addition to reducing the possibility of organ rejection, MMF blocked the expansion of B lymphocytes infected by the EBV [160]; exerted antiviral action against HIV [161], HBV [162], and HEV [32]; or augmented the effects of other approved antiviral agents to treat various chronic viral diseases associated with liver [163,164,165,166] or renal dysfunction [167].

### 5.6. Merimepodib

Merimepodib (MMPD, VX-497) is a potent, reversible, and uncompetitive *in vitro* and *in vivo* IMPDH inhibitor that binds to the enzyme–substrate complex. Structurally it is unrelated to other IMPDH inhibitors and was developed through a structure-based drug design program carried out by Vertex Pharmaceuticals [168]. Initially intended for the treatment of various disorders/diseases where immunosuppression could generate a benefit, it was later evaluated for its antiviral action against various DNA and RNA viruses [169]. It advanced to phase 2b of clinical trials in humans against HCV but was terminated because it showed modest benefits compared to existing approved treatments even in combination therapy [170]. More recently, the antiviral spectrum has been broadened, showing inhibitory action *in vitro* against several other emerging viral pathogens [171]. 

### 5.7. New IMPDH Inhibitors

IMPDH inhibitors were recently designed with the aim of establishing their activity towards this enzyme and improving the therapeutic properties of inhibitors used as anticancer, antiviral, antibacterial, and immunosuppressive drugs. Many of these new compounds and analogs exhibited promising results during *in vitro* and *in vivo* tests compared to clinically applied substances [172,173].

Compounds that exhibited potent broad-spectrum antiviral activity at low micromolar concentrations emerged from a series of novel unsaturated benzo heterocyclic amine derivatives evaluated against a wide range of DNA and RNA genome viruses. These derivatives with electron-withdrawing substituents on the aromatic or heteroaromatic rings showed a greater effect against RNA viruses [174]. One of them, N30, provided strong inhibition of the *in vitro* replication of oseltamivir-resistant and amantadine-resistant strains of IAV, RSV, coronavirus, EV71, and various strains of coxsackie B virus (CBV) by suppressing the activity of IMPDH type II [175].

Compounds with a distinct chemical moiety that differ from other reported IMPDH inhibitors could also be identified through a high-throughput screening system or in silico docking studies. The analysis of a chemical library of 1400 known small bioactive molecules resulted in the discovery of three irreversible inhibitors: disulfiram, bronopol, and ebselen. These compounds are potent inhibitors of protozoan and mammalian IMPDHs. Disulfiram and bronopol were active *in vivo* against cryptosporidiosis in mice with severe combined immunodeficiency, providing clinical proof of concept for the study of these compounds as inhibitors of IMPDH [176]. On the other hand, using a JUNV minigenome luciferase-based system that provides a platform for the detection of factors that only affect RNA synthesis, a limited screening of a drug library was performed to identify AVN-944 (VX-944, Avalon Pharmaceutical), a non-competitive non-nucleoside inhibitor of IMPDH [177]. The antiviral effect of AVN-944 was confirmed against *in vitro* infection with New World arenaviruses [178]. In an influenza virus minigenome assay, the non-nucleoside compound CPD A (N-(pyridin-3-yl) thiophene-2-carboxamide) inhibited RNA synthesis of a broad panel of IAV (H1N1 and H3N2) and IBV strains through a strong depletion of the cellular GTP pool. CPD A required metabolic activation since no direct inhibition was seen in an enzymatic IMPDH assay and its combination with RIB proved strong synergism [179]. Additionally, molecular docking studies were carried out that allowed the identification of highly potent and selective inhibitors of several bacterial/protozoal IMPDH. Such studies reveal the importance of this excellent target for the treatment of infectious diseases and its potential extension to other pathologies [180].

## 6. Concluding Remarks and Perspectives

Diverse properties have been attributed to the inhibitors of nucleotide biosynthesis through the depletion of the intracellular nucleotide pool such as the blockade of nucleic acid synthesis, induction of an antiviral innate immune response, and arresting of the inflammatory cytokine expression. Altogether, the combination of this set of activities highlights the antiviral potential of these HTA against pathogenic viruses. In fact, compounds affecting DHODH and IMPDH, key enzymes for pyrimidine and purine biosynthesis, respectively, were found to be effective inhibitors of a wide spectrum of viruses affecting human health. 

However, there was not total correspondence between the *in vitro* and *in vivo* antiviral efficacy of these compounds when tested in animal models or clinical trials. This controversy could be due to the high level of pre-existing nucleosides in plasma compared to cell culture conditions. Therefore, the efficiency of the salvage pathway for recycling nucleotide precursors leads to a bypass of the blockade in the *de novo* synthesis reducing the antiviral potential of IMPDH or DHODH inhibitors in the clinic. Furthermore, the greater use of the salvage pathway appears not to affect the uridine levels present as a reservoir of nucleic acid precursors. The combination of the inhibitors of both the *de novo* and salvage pathways is a strategy under development to confront this inconvenience. Moreover, increased antiviral activity was demonstrated for the simultaneous use of *de novo* inhibitors with DAA-like nucleoside analogs. A synergistic response was obtained because compounds are directed to differential targets and also the undesirable side effects produced by both agents were diminished. Additionally, it is speculated that the reduction in purine/pyrimidine levels produced by *de novo* inhibitors seems to promote higher incorporation of nucleoside analogs in the growing chain of nucleic acid. 

In summary, the search for more potent *de novo* nucleotide biosynthesis inhibitors is under development in order to obtain a wide spectrum of antiviral agents for the treatment of emerging and re-emerging viruses. New avenues in this field have been explored with the intention of combining therapies to improve their potential for clinical application.

## Figures and Tables

**Table 1:** Efficacy and antiviral spectrum of the DHODH inhibitors.

DHODH Inhibitor	Viral Model	Efficacy [EC50]	Ref.		DHODH Inhibitor	Viral Model	Efficacy [EC50]	Ref.
**Leflunomide**	BKV	40 µg/mL	[54]		**Vidofludimus calcium (IMU-838)**	SARS-CoV-2	3.2–7.6 µM	[77]
	EBV	10 µg/mL	[55]			HCMV	7.4 µM	[77]
	SARS-CoV-2	41.49–48.98 µM	[34]			HCV	4.5 µM	[77]
						HIV-1	2.1 µM	[77]
**Teriflunomide** **(A77 1726)**	HCMV	40–60 µM	[53]		**A3**	IAV	170 nM	[78]
	JUNV	16–45 µM	[57]			HIV-1	205 nM	[78]
	IAV	2.73–35.02 µM	[58]			HCV	<2 µM	[78]
	ZIKV	17.72 µM	[34]			AdV-5	<2 µM	[78]
	EBOV	3.41 µM	[34]			LCMV	82 nM	[79]
	SARS-CoV-2	6.0–26.06 µM	[34]			JUNV	<1 µM	[79]
**Brequinar**	WNV	3 µM	[66]		**FA-613**	IAV	3.8 µM	[30]
	YFV	3 µM	[66]			IBV	0.2 µM	[30]
	DENV	78 nM	[66]			EA71	8.6 µM	[30]
	ZIKV	0.3–1.51 µM	[20]			RSV	10.1 µM	[30]
	EBOV	0.1 µM	[34]			MERS-CoV	>30 µM	[30]
	HCMV	17 nM	[67]			SARS-CoV-1	>30 µM	[30]
	IAV	0.24 µM	[34]			Rhinovirus AV	9.7 µM	[30]
	EV71	82.40 nM	[68]		**MEDS433**	HSV-1	78–116 nM	[83]
	EV70	29.26 nM	[68]			HSV-2	61–95 nM	[83]
	CVB3	35.14 nM	[68]			HCoV-OC43	12 nM	[84]
	RV	49.17 nM	[69]			HCoV-229E	22 nM	[84]
	SARS-CoV-2	0.06–0.794 µM	[34,70]			SARS-CoV-2	63–76 nM	[84]
**S312/S416**	IAV	0.013–13.7 µM	[34]					
	ZIKV	0.019–1.24 µM	[34]		**RYL-634**	HCV	1.56 µM	[85]
	EBOV	0.018–11.39 µM	[34]			DENV	7 nM	[85]
	SARS-CoV-2	0.014–1.59 µM	[34]			ZIKV	20 nM	[85]
**Emvododstat (PTC299)**	SARS-CoV-2	2.0–31.6 nM	[35]			EV71	4 nM	[85]
	EBOV	9.1 nM	[35]			HIV	13 nM	[85]
	PV	0.57 nM	[35]			IAV	<1 µM	[85]
	HCV	36 nM	[35]		**AR-12 derivatives**	DENV	62.2–68 nM	[87]
	RVFV	13 nM	[35]			HIV	118.6–130.3 nM	[87]
**BAY2402234**	SARS-CoV-2	5–11nM	[70]			IAV	53.2–56.1 nM	[87]
**GSK983/SW835**	EBOV	0.009–1 µM	[31]					

EC50 (effective concentration 50%): concentration required to reduce virus infection by 50%.
